# Ent-11α-hydroxy-15-oxo-kaur-16-en-19-oic-acid induces apoptosis and cell cycle arrest in CNE-2Z nasopharyngeal carcinoma cells

**DOI:** 10.3892/or.2013.2375

**Published:** 2013-04-03

**Authors:** KEFENG WU, YI LIU, YINGNIAN LV, LIAO CUI, WENDE LI, JIANFA CHEN, NIAN CI LIANG, LI LI

**Affiliations:** 1 Guangdong Key Laboratory for Research and Development of Natural Drugs, Guangdong Medical College, Zhanjiang, Guangdong 524023, P.R. China; 2 Department of Surgery, Prince of Wales Hospital, The Chinese University of Hong Kong, New Territories, Hong Kong, SAR, P.R. China; 3 Department of General Surgery, 422 Hospital of PLA, Zhanjiang, Guangdong 524009, P.R. China; 4 Institute of Biochemistry and Molecular Biology, Guangdong Medical College, Zhanjiang, Guangdong 524023, P.R. China

**Keywords:** ent-11α-hydroxy-15-oxo-kaur-16-en-19-oic-acid, nasopharyngeal carcinoma, p53, NF-κB, cisplatin

## Abstract

Ent-11α-hydroxy-15-oxo-kaur-16-en-19-oic-acid (5F), a compound isolated from *Pteris semipinnata* L. (PsL), inhibits cell proliferation and induces cell apoptosis in several cancer lines. We found that 5F induced apoptosis and G2 phase cell cycle arrest in the CNE-2Z nasopharyngeal carcinoma (NPC) cells, accompanied by a decrease of NF-κB expression. 5F suppressed the viability of CNE-2Z cells in a time- and dose-dependent manner. 5F induced G2/M phase cell cycle arrest, but did not induce p21. Further analysis revealed that CNE-2Z cells harbored two p53 mutations. 5F treatment resulted in mitochondrial apoptosis, associated with increased Bax/Bcl-2 ratio, upregulation of cytochrome *c* in the cytosol, decreased NF-κB-p65 and increased IκB. Of note, 5F significantly sensitized CNE-2Z cells to cisplatin. 5F did not increase ROS, but reduced ROS production alone or in combination with cisplatin. Our data suggest that 5F is a potential anti-NPC drug for the development of single agent therapy and therapy in combination with cisplatin.

## Introduction

The incidence rate of nasopharyngeal carcinoma (NPC) in men is higher in rural than in urban China. NPC is the seventh most common cancer in men in rural areas with an incidence rate of 6.08/100,000, followed by bladder cancer, brain cancer and lymphoma (1). Despite significant advances in the therapy and early diagnosis, the prognosis of patients with stage III and IV NPC remains poor, usually due to a relatively high incidence of locoregional recurrence or metastasis (2). Thus, there is an urgent need to develop effective and safe therapeutics for this malignancy.

Ent-11α-hydroxy-15-oxo-kaur-16-en-19-oic-acid (5F) is an active compound in *Pteris semipinnata* L (PsL). *In vitro*, 5F has been shown to kill various human cancer cells including lung cancer cells, laryngeal cancer cells, thyroid carcinoma cells, gastric cancer cells and colorectal cancer cells via apoptotic pathways (3–7). Since 5F can induce apoptosis in cells of various types of cancer, it may also be a potential apoptosis-inducing drug in NPC therapy. In the present study, we examined the potential antitumor action of 5F in a human NPC cell line, CNE-2Z, and explored the possible synergism between 5F and cisplatin.

## Materials and methods

### Cell culture

CNE-2Z is a poorly differentiated human NPC cell line (8). CNE-2Z cells were cultured in RPMI-1640 medium (Sigma-Aldrich) containing 10% fetal bovine serum (FBS) (Sigma-Aldrich), 100 U/ml penicillin and 100 μg/ml streptomycin. Cells were cultured at 37°C in a humidified 5% CO_2_ incubator.

### Mutational analysis of p53

Genomic DNA was extracted from CNE-2Z using a genomic DNA isolation kit (Sangon, Shanghai, China) following the manufacturer’s instructions, and quantified using a NanoDrop spectrophotometer (NanoDrop Technologies, Wilmington, DE, USA). The primers for PCR are listed in Table I. A 300 ng aliquot of genomic DNA was added to a PCR master mixture containing 1X PCR buffer (100 mM Tris-HCl, 500 mM KCl, pH 8.3), 200 μM each of deoxynucleoside triphosphate, 200 nM primer, 1.5 mM MgCl_2_, and 2.5 units of Taq polymerase. PCR was performed under the following cycling conditions: 4 min at 95°C, followed by 45 sec at 94°C, 45 sec at 68°C, 1 min at 72°C for 35 cycles and final extension for 8 min at 72°C. The PCR amplicons were purified with Gel/PCR DNA Fragments Extraction kit (Sangon) and sequenced with the BigDye Terminator kit (Applied Biosystems, Foster City, CA, USA) and ABI Prism 3730 DNA Analyzer (Applied Biosystems) according to the manufacturer’s instructions.

### Ent-11α-hydroxy-15-oxo-kaur-16-en-19-oic-acid (5F)

**Figure 1:**
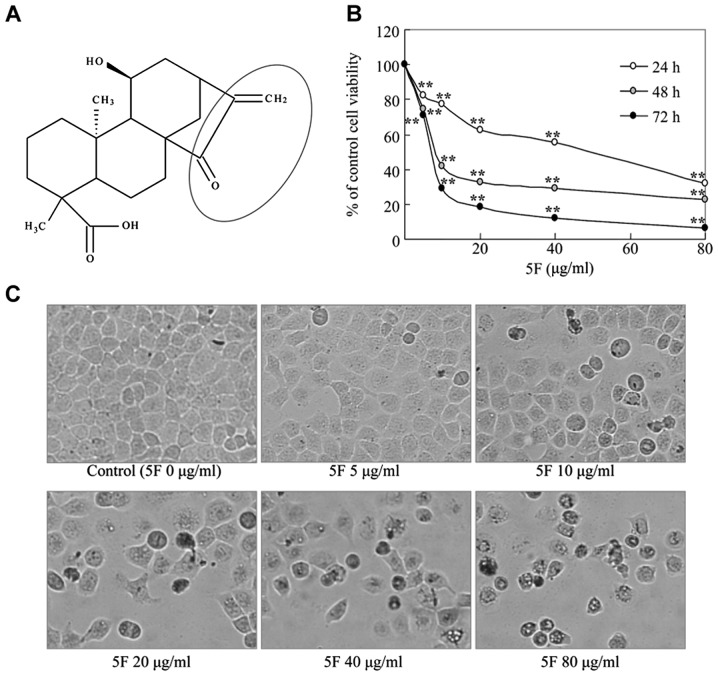
5F inhibits the growth of CNE-2Z nasopharyngeal carcinoma cells. (A) Chemical structure of 5F. The circle indicates the conjugated double bonds structure. (B) Cell viability was examined following treatment with different concentrations of 5F as indicated for 24, 48 or 72 h using MTT assay. Drug effect was expressed as percentage relative to the controls (^*^P<0.05, ^**^P<0.01). (C) Morphology of the cells was examined 24 h after 5F exposure under an inverted phase contrast microscope (magnification, ×100).

5F was isolated from PsL as previously described (9) (Fig. 1A). 5F was dissolved in propylene glycol (PG) and diluted with the culture medium immediately prior to use (final PG concentration ≤1.2%). In all experiments, the cells in RPMI-1640 medium plus PG only were used as the control.

### Cell viability assay

CNE-2Z cells (5×10^3^) were seeded in each well of a 96-well plate in 200 μl medium. At 24 h, the cells were exposed to 5F (0–80 μg/ml), cisplatin (10 μg/ml), or a combination of 5F (10 μg/ml) and cisplatin (10 μg/ml). Cell viability was examined after an additional 24, 48 or 72 h using MTT assay. Drug effect was expressed as percentage relative to the controls. Morphology of the cells was examined 24 h after 5F exposure under an inverted phase contrast microscope.

### DAPI staining assay

Cells were fixed with 4% paraformaldehyde for 20 min, washed with PBS, and then incubated with DAPI (2 μg/ml) (Beyotime Institute Biotechnology, Haimen, China) at room temperature (RT) for 10 min. Following removal of free DAPI with PBS, cells were observed under a fluorescence microscope.

### Cell cycle distribution

CNE-2Z cells were seeded at a density of 1×10^5^ cells/well in a 6-well plate. Following treatment, cells were fixed overnight with 70% ethanol at −20°C and stained with PI solution (100 μg/ml) (Sigma-Aldrich). Cell cycle distribution analysis was performed using a flow cytometer.

### Measurement of apoptosis

Following treatment, cells were harvested, washed with PBS and resuspended in 195-μl binding buffer. The samples were then incubated with 5 μl Annexin V-FITC for 15 min in the dark at 4°C and subjected to flow cytometry analysis immediately. Data acquisition and analysis were performed on a Becton-Dickinson FACSCalibur flow cytometer using CellQuest software. Caspase-3 activity was measured using Caspase-3 Colorimetric Assay kit (Nanjing KeyGen Biotech. Co., Ltd., Nanjing, China) according to the manufacturer’s instructions. Caspase-3 activity was expressed as: OD_test compound_/OD_control_.

### Reactive oxygen species (ROS) generation

The intracellular ROS level was measured using a fluorescent dye DCFH-DA (ROS Assay kit, Beyotime Institute Biotechnology). Cells were exposed to 5F (0 and 10 μg/ml), cisplatin (10 μg/ml), or a combination of 5F (10 μg/ml) and cisplatin (10 μg/ml) for 3 h, and incubated in the dark with 10 μM DCFH-DA in serum-free medium for 20 min at 37°C. ROS generation was detected using a FACScan flow cytometer with the excitation and emission settings at 488 and 530 nm, respectively.

### Western blot analysis

Cells were lysed on ice in SDS Lysis Buffer (Beyotime Institute Biotechnology) supplemented with protease inhibitors and phosphatase inhibitor (Roche), and 1 mM PMSF (Sigma). Cytoplasmic extract was obtained using a Cytoplasmic Protein Extraction kit (Beyotime Institute Biotechnology). The protein concentration was determined using a BCA Protein Assay kit (Beyotime Institute Biotechnology). Immunoblots of 50 μg total protein were probed with the following antibodies: cytochrome *c*, IκB, actin (Santa Cruz Biotechnology, Santa Cruz, CA, USA), Bcl-2, Bax (Zhongshan Golden Bridge Biotechnology, Wuhan, China), p21 (Boster Bio-Engineering Ltd., Wuhan, China), NF-κB-p65 (Phospho-Ser536) (SAB Signalway Antibody, Pearland, TX, USA). Protein bands were visualized using chemiluminescent reagents.

### Statistical analysis

Data are presented as the means ± SD. The differences between the groups were examined with one-way analysis of variance (ANOVA) using the SPSS 13 software (SPSS Inc., Chicago, IL, USA). P<0.05 was considered to indicate a statistically significant difference.

## Results

### 5F inhibits the proliferation of CNE-2Z NPC cells

*In vitro*, 5F has been shown to inhibit cell viability of various human cancer cells (3–7). To test if 5F prevents the proliferation and growth of NPC cells, we treated CNE-2Z NPC cells with different concentrations of 5F for 24, 48 or 72 h and examined the cell proliferation using a standard MTT method. 5F treatment led to a time- and dose-dependent decrease of the cell viability of CNE-2Z cells (Fig. 1B). Moreover, 5F-treated cells exhibited a rounded and granulated morphology, and were detached from culture wall after a 24-h exposure (Fig. 1C). Thus, 5F also inhibits the proliferation and growth of CNE-2Z NPC cells.

### 5F induces apoptosis of CNE-2Z cells

**Figure 2:**
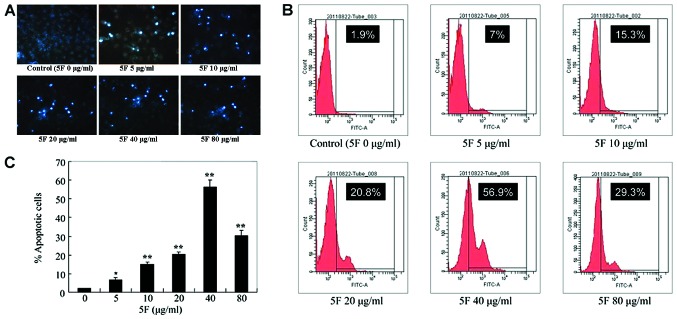
5F induces apoptosis of CNE-2Z cells. (A) Cells were fixed with 4% paraformaldehyde for 20 min, washed with PBS, and then incubated with DAPI (2 μg/ml) at room temperature for 10 min (magnification, ×100). (B) Following treatment with 5F for 24 h, cells were harvested, washed with PBS, resuspended in binding buffer and then incubated with Annexin V-FITC for 15 min in the dark at 4°C and subjected to flow cytometry analysis. (C) Quantification of (B). ^*^P<0.05, ^**^P<0.01.

To test whether 5F inhibits the cell growth of CNE-2Z cells by inducing apoptosis, we stained 5F-treated cells with the fluorescent dye DAPI and observed nucleus changes under the fluorescence microscope. Compared with untreated cells, 5F-treated cells demonstrated bright nuclear condensation, nuclear pyknosis and apoptotic bodies, indicating that 5F induces apoptosis in CNE-2Z cells (Fig. 2A). To extend our observation, we further stained 5F-treated cells with Annexin V-FITC, an early indicator of apoptosis. As shown in Fig. 2B and C, 5F treatment of CNE-2Z cells resulted in a 5.1, 13.4, 18.9, 55 and 27.4% increase of Annexin V-positive cells at 5, 10, 20, 40 and 80 μg/ml 5F, respectively. These results clearly show that 5F induces significant apoptosis in CNE-2Z cells.

### 5F induces G2 cell cycle arrest in CNE-2Z cells independently of p53-p21 axis

**Figure 3:**
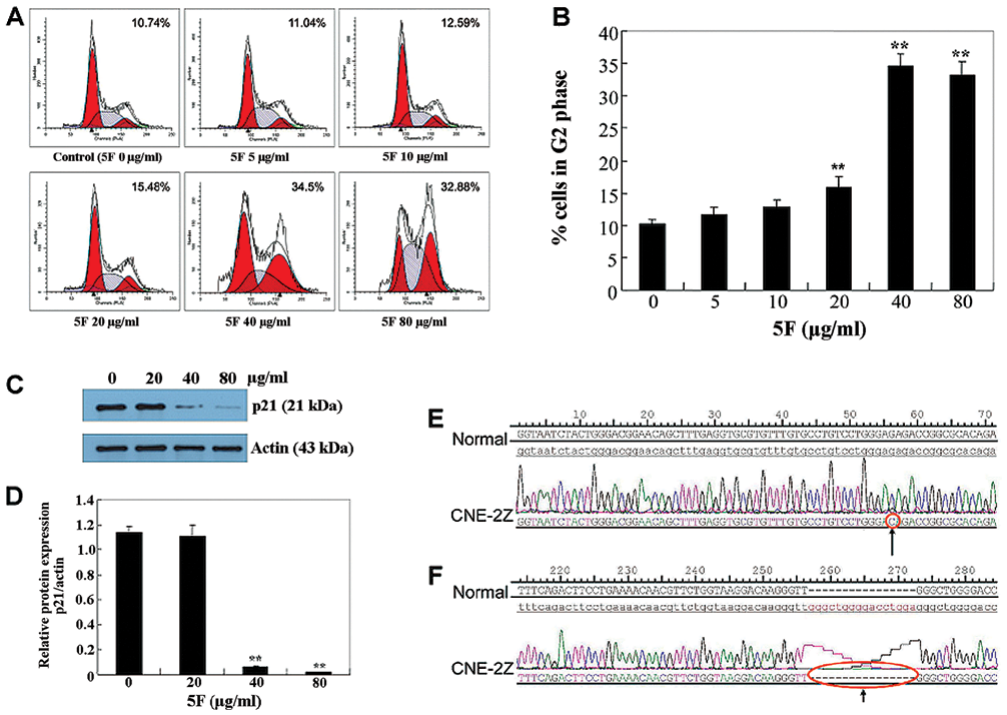
5F induces G2 cell cycle arrest in CNE-2Z cells independently of p53-p21 axis. (A) Following treatment with 5F for 24 h, cells were fixed overnight with 70% ethanol at −20°C and stained with PI solution. Cell cycle distribution analysis was performed using a flow cytometer. (B) Representative cell cycle histogram of (A) demonstrating the percentage of the cells in the G2 phase. Compared with blank control ^**^P<0.01). (C) Effect of 5F on p21 expression, as measured by western blot analysis. Compared with blank control (^**^P<0.01). (D) Quantification of (C). ^**^P<0.01. (E) Sequencing analysis shows (arrows) a G-to-C change, at position 56 of exon 8 of the TP53 gene. (F) Sequencing analysis shows (arrows) a deletion of gggctggggacctgga in the position 16–31 of intron 3 of the TP53 gene.

It has been well documented that 5F induces G2 phase of cell cycle arrest in several cell lines. To determine whether the growth-inhibitory effect of 5F in CNE-2Z cells is associated with the induction of cell cycle arrest, we analyzed the distribution of cells in the different phases of the cell cycle using flow cytometry. Following treatment with 5, 10, 20, 40 and 80 μg/ml 5F for 24 h, the percentage of cells in the G2/M phase was 11.04, 12.59, 15.48, 34.5 and 32.88%, respectively (Fig. 3A and B), indicating that 5F induces cell cycle arrest in the G2 phase in CNE-2Z cells.

To investigate the mechanism by which 5F causes the G2-phase cell cycle arrest, we examined the effects of 5F on the expression of p21, which regulates the G2-phase checkpoint (10–12). Notably, 5F significantly reduced p21 protein levels at 40 and 80 μg/ml (Fig. 3C and D). p21 is the critical downstream transcriptional target of p53, the reduction but not the increase of p21 by 5F suggests that there may be a p53 mutation in CNE-2Z cells. Therefore, we sequenced the gene of p53 of CNE-2Z cells and found two types of changes. The first was a G-to-C change, at position 56 in exon 8 (Fig. 3E), and the other was a deletion of GGGCTGGGGACCTGGA in the position 16–31 in intron 3 (Fig. 3F). Thereafter, the failure to induce p21 by 5F is likely due to the loss-of-function of p53.

### 5F reduces intracellular ROS levels

**Figure 4:**
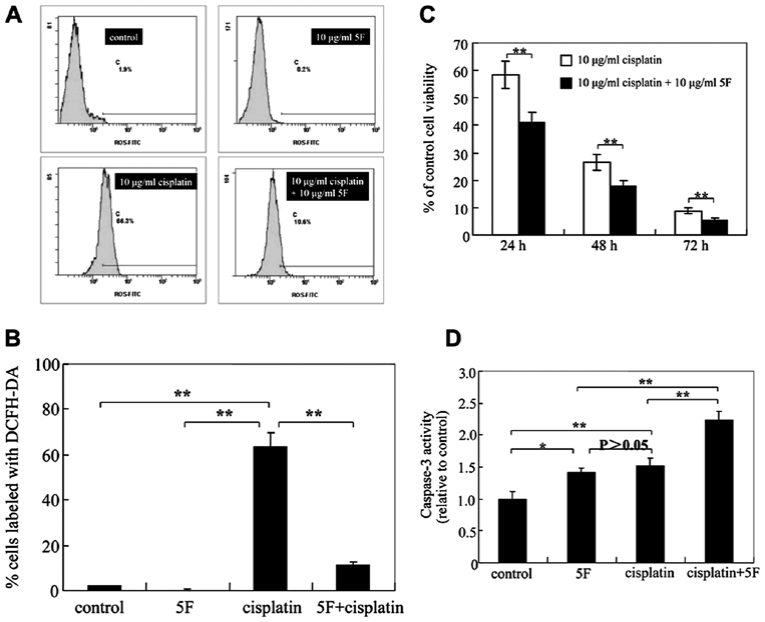
5F reduces intracellular ROS levels and sensitizes CNE-2Z cells to cisplatin. (A) Cells were exposed to 5F, cisplatin, or a combination of 5F and cisplatin for 3 h, and incubated in the dark with 10 μM DCFH-DA for 20 min at 37°C. ROS generation was detected using a FACScan flow cytometer. (B) Quantification of (A). ^**^P<0.01. (C) Cells were treated with 5F, cisplatin, or a combination of 5F and cisplatin for 24, 48 and 72 h. MTT assays were used to check the viability. ^**^P<0.01. (D) Cells were treated as in (A). Caspase-3 activity was measured using Caspase-3 Colorimetric assay. Caspase-3 activity was expressed as: OD_test compound_/OD_control_. ^*^P<0.05, ^**^P<0.01.

ROS induces endogenous DNA damages and plays an important role in apoptosis in a variety of cells. To test the possibility that 5F induces cell apoptosis and G2 phase arrest by inducing ROS generation in CNE-2Z cells, we measured intracellular ROS levels. As shown in Fig. 4A and B, 5F significantly decreased ROS generation in CNE-2Z cells for 3 h. Thus, 5F reduces intracellular ROS levels and the induction of the G2 phase might not be due to the ROS-induced DNA damages.

### 5F sensitizes CNE-2Z cells to cisplatin independently of its reduction of ROS

**Figure 5:**
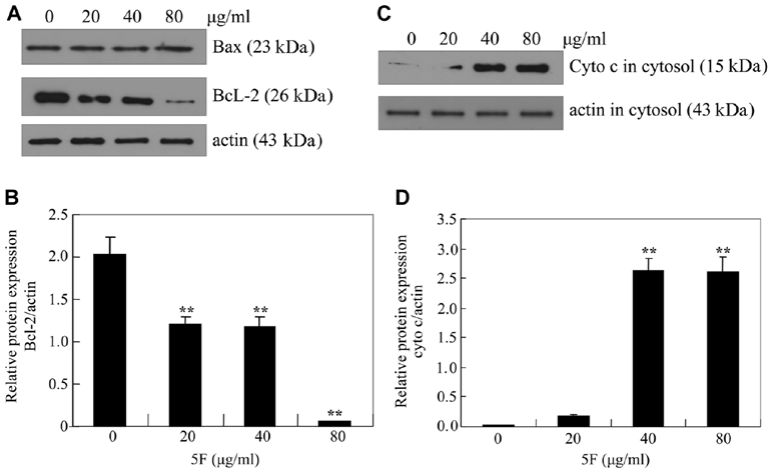
5F downregulates the ratio of Bcl-2/Bax. (A) Cells were treated with different concentrations of 5F as indicated for 24 h. Bax and Bcl-2 proteins were measured by western blot analysis. (B) Quantification of Bcl-2 relative to actin of (A). ^**^P<0.01. (C) Cells were treated as in (A). Effects of 5F on the release of cytochrome *c* from mitochondria into the cytosol were measured by western blot analysis. (D) Quantification of (C). ^**^P<0.01.

It has been reported that cisplatin cytotoxicity is mediated by increased generation of ROS. Several reports have demonstrated that cisplatin-induced cytotoxicity could be ameliorated by using antioxidants or oxygen radical scavengers (13–15). The reduction of ROS by 5F predicts that 5F may attenuate cisplatin cytotoxicity. We thus determined whether 5F antagonizes cisplatin-stimulated intracellular ROS generation. We found that cisplatin increased ROS production (Fig. 4A and B) and 5F reduced the ROS production in cisplatin-treated CNE-2Z cells, whereas 5F significantly sensitized CNE-2Z cells to cisplatin-induced cytotoxicity after 24, 48 and 72 h of treatment (Fig. 4C). Moreover, a synergistic interaction in increasing caspase-3 activity was also observed when 5F (10 μg/ml) was added to 10 μg/ml of cisplatin in CNE-2Z NPC cells (Fig. 4D). Thus, 5F sensitizes CNE-2Z cells to cisplatin via the apoptosis pathway.

### 5F downregulates Bcl-2 and upregulates IκBα

To elucidate the mechanism by which 5F induces apoptosis in CNE-2Z cells, we measured the protein levels of Bcl-2 and Bax. Treatment of CNE-2Z cells with 5F resulted in a dose-dependent decrease of anti-apoptotic Bcl-2 protein. By contrast, 5F did not significantly influence the expression of the pro-apoptotic Bax protein (Fig. 5A and B). Accordingly, 5F induced a dose-dependent release of cytochrome *c* from mitochondrion (Fig. 5C and D). These data indicated that 5F induces the activation of mitochondrial-mediated internal apoptosis pathway by downregulating Bcl-2.

**Figure 6:**
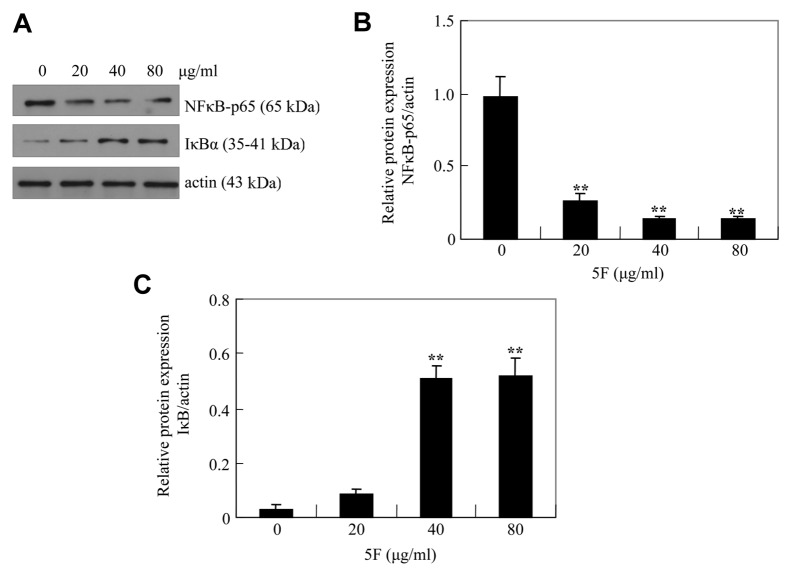
5F increases IκBα and decreases NF-κB-p65. (A) Cells were treated with different concentrations of 5F as indicated for 24 h. NF-κB-p65 and IκB proteins were measured by western blot analysis. (B) Quantification of NF-κB-p65 relative to actin. ^**^P<0.01. (C) Quantification of IκB relative to actin. ^**^P<0.01.

Bcl-2 is regulated by nuclear factor κB (NF-κB). To test if 5F induces apoptosis by downregulating Bcl-2 through NF-κB, we examined p65, a subunit of NF-κB, and IκBα, an inhibitor of NF-κB (16). We found that the level of p65 was reduced by 5F whereas the level of IκBα was upregulated by 5F (Fig. 6). These results suggest that 5F might induce apoptosis of CNE-2Z cells by downregulating Bcl-2 by decreasing NFκB signaling.

## Discussion

In the present study, we demonstrated that 5F significantly inhibited the proliferation of CNE-2Z cells in a dose- and time-dependent manner, and this inhibitory effect was p53 independent. Moreover, 5F induced mitochondrial-mediated apoptosis in CNE-2Z cells, accompanied by a decrease of Bcl-2 and NF-κB. Finally, we found that 5F sensitized CNE-2Z cells to cisplatin independently of its reduction of ROS. Our data suggest that 5F may be a potential anti-NPC agent.

Deregulation of cell cycle progression and evasion of apoptosis are hallmarks of cancer cells (17). Accordingly, inhibition of cell cycle progression may be particularly useful in the treatment of cancer. 5F has been demonstrated to arrest cells at the G2 phase in FRO cells (an anaplastic thyroid carcinoma cell line) and A549 cells (a non-small cell lung cancer cell line) (5,3). Consistent with these reports, the present study showed that 5F could induce G2-phase arrest in CNE-2Z cells. The G2 phase of the cell cycle is controlled by cyclin-dependent kinase 1 (CDK1) and can be inhibited by upregulation of the cyclin-dependent kinase (CDK) inhibitor p21 (18). Several studies have shown that anticancer drugs can increase the number of cells in G2/M cell cycle phases, accompanied by upregulation of p21 (19–23). Therefore, we measured the expression of p21 in CNE-2Z cells to further investigate the reason for the G2/M-phase arrest mediated by 5F. Markedly, our results showed that 5F reduced the expression of p21 in CNE-2Z cells. In fact, the functions of p21 are very complex. Despite the critical role of p21 in arresting cell cycle progression and promoting differentiation and senescence, it was shown that p21 could also promote cellular proliferation and tumorigenesis under certain conditions. Consequently, depending on the cellular context and circumstances, p21 is often deregulated in human cancer, suggesting that it can act either as a tumor suppressor or as an oncogene (24). The tumor suppressor p53 is a nuclear transcription factor that induces the expression of its numerous downstream targets including p21, leading to cell cycle arrest, senescence and apoptosis (25). In the present study, we found p53 is a mutant in CNE-2Z cells. The reduction of p21 by 5F in CNE-2Z cells may be partly due to the p53 mutation.

Previous reports have shown that several antitumor agents (such as cisplatin) arrest cell cycle at the G2/M phase, accompanied by apoptosis (26). Induction of tumor cell death by apoptosis is a major mechanism employed by antitumor agents. In the present study, we demonstrated apoptosis-inducing effects of 5F in CNE-2Z cells using DAPI staining and Annexin V-FITC staining assay. The mitochondrial-mediated apoptotic pathway contributes to apoptosis of cancer cells induced by several antitumor agents (27–29). Elevated ratio of Bax/Bcl2 is an important marker of apoptosis in several cancer cells (23,30–33). Based on the quantification of western blot signals by densitometry, an approximately 23-fold increase of Bax/Bcl2 was observed following treatment with 80 μg/ml of 5F for 24 h (Fig. 5A and B). In addition, the release of cytochrome *c* from mitochondria into the cytosol was also observed. These results suggest that 5F-induced apoptosis in CNE-2Z cells is mediated, at least in part, by the mitochondrial pathway. On the other hand, caspase-3 plays a pivotal role in the terminal execution phase of apoptosis (34). An increasing caspase-3 activity was observed when 10 μg/ml of 5F was added to 10 μg/ml of cisplatin in CNE-2Z cells, which suggests that the sensitization of CNE-2Z cells by 5F to cisplatin is associated with enhanced apoptosis.

The transcription factor NF-κB is an important mediator of cell cycle progression and cell survival associated with carcinogenesis (35). Upregulation of NF-κB is positively associated with poor outcome of several types of cancer, including NPC (36,37). In resting cells, NF-κB exists in the cytoplasm and is inhibited by complexing with IκB. Phosphorylation of IκB by IκB kinase (IKK) causes ubiquitination and degradation of IκB. Subsequently, NF-κB is released and translocates to the nucleus, where NF-κB regulates the transcription of a number of genes which are involved in tumorigenesis and cell growth (38,35). These findings indicate that NF-κB is a potential therapeutic target in cancer. In the present study, we investigated the effects of 5F on the pattern of NF-κB activation. Our results showed that treatment of CNE-2Z cells with 5F significantly inhibited the expression of p-NF-κB/p65 protein and degradation of IκBα protein. This study suggested that the effects of 5F on NF-κB/p65 might be through inhibition of the phosphorylation and subsequent proteolysis of IκBα.

Several antitumor agents such as cisplatin, adriamycin (ADR), bleomycin and tumor necrosis factor (TNF) have been reported to exert cytotoxic effects through ROS induction (39–42), therefore, we determined the role of ROS formation in 5F-induced cell death in CNE-2Z cells. Notably, 5F induced apoptosis and, at the same time, reduced the ROS generation in CNE-2Z cells. ROS scavenging of 5F might be related to conjugated double bonds. Cisplatin is one of the most effective chemotherapeutic agents, but has prominent side-effects such as nephrotoxicity. These side-effects are believed to be related to its ability to induce ROS production (43,44). Animal studies have indicated that antioxidants or oxygen radical scavengers could either ameliorate or protect against cisplatin toxicity (44,45). Results from the current study demonstrated that 5F possesses antioxidant properties in addition to anticancer properties. Therefore, 5F combined with cisplatin might improve cisplatin anticancer effects by reducing the cisplatin-induced ROS.

In conclusion, 5F inhibited CNE-2Z cell proliferation through cell cycle arrest at the G2/M phase and apoptosis induction related to the mitochondrial-mediated apoptotic pathway and NF-κB inhibition. Our results also indicate that 5F sensitizes CNE-2Z cells to cisplatin-induced cytotoxicity and reduces ROS production induced by cisplatin. Our previous studies in mice showed that 5F could be effective against liver and lung cancer with minimal side-effects (46,47). These results suggest 5F is a promising anti-NPC agent.

## Figures and Tables

**Table I:** Primer sequences used to amplify p53 tumor suppressor gene.

Exons	Primer sequences (5′-3′)	Product size (bp)
2–4	F: GAAGTCTTGGGGATTTAGTGGT R: CAAAAGCCAAGGAATACACG	1192
5–6	F: GGTTGCAGGAGGTGCTTACG R: GTTTCACCGTTAGCCAGGAT	991
7	F: AGGCTGAGGAAGGAGAATGG R: GGGTAGTAGTATGGAAGAAATCGG	406
8–9	F: AGGGTGGTTGGGAGTAGATG R: GCAGGCTAGGCTAAGCTATGAT	791
10	F: GAGGCTGAGGCACAAGAATC R: CCCTGGGTTTGGATGTTCTG	601
11	F: GCAACAAGAGTGAAACTCCGT R: TTACATCTCCCAAACATCCCT	594

F, forward; R, reverse.
